# Severe Post-COVID-19 Condition after Mild Infection: Physical and Mental Health Eight Months Post Infection: A Cross-Sectional Study

**DOI:** 10.3390/ijerph21010021

**Published:** 2023-12-22

**Authors:** Marion Egger, Lena Vogelgesang, Judith Reitelbach, Jeannine Bergmann, Friedemann Müller, Klaus Jahn

**Affiliations:** 1 Research Group, Department of Neurology, Schoen Clinic Bad Aibling, 83043 Bad Aibling, Germany; 2 Institute for Medical Information Processing, Biometry, and Epidemiology (IBE), Faculty of Medicine, LMU Munich, Pettenkofer School of Public Health, 81377 Munich, Germany; 3 German Center for Vertigo and Balance Disorders, University Hospital Grosshadern, Ludwig-Maximilians-Universität (LMU), 81377 Munich, Germany

**Keywords:** COVID-19, post-acute COVID-19 syndrome, critical illness, neurological rehabilitation, quality of life, fatigue, mental health, patient reported outcome measures, hospitalization, intensive care units

## Abstract

Severe acute COVID-19 infections requiring intensive care treatment are reported risk factors for the development of post-COVID-19 conditions. However, there are also individuals suffering from post-COVID-19 symptoms after mild infections. Therefore, we aimed to describe and compare the health status of patients who were initially not hospitalized and patients after critical illness due to COVID-19. The outcome measures included health-related quality of life (EQ-5D-5L, visual analogue scale (VAS)); mental health (hospital anxiety and depression scale (HADS)); general disability (WHODAS-12); and fatigue (Fatigue-Severity-Scale-7). Individuals were recruited at Schoen Clinic Bad Aibling, Germany. A total of 52 non-hospitalized individuals (47 ± 15 years, 64% female, median 214 days post-infection) and 75 hospitalized individuals (61 ± 12 years, 29% female, 235 days post-infection) were analyzed. The non-hospitalized individuals had more fatigue (87%) and anxiety (69%) and a decreased health-related quality of life (VAS 47 ± 20) compared to the hospitalized persons (fatigue 45%, anxiety 43%, VAS 57 ± 21; *p* < 0.010). Severe disability was observed in one third of each group. A decreased quality of life and disability were more pronounced in the females of both groups. After adjusting for confounding, hospitalization did not predict the burden of symptoms. This indicates that persons with post-COVID-19 conditions require follow-up services and treatments, independent of the severity of the acute infection.

## 1. Introduction

The SARS-CoV-2 pandemic caused millions of infections all over the globe. Not only did the COVID-19 disease lead to millions of deaths, it has also led to a substantial group of individuals suffering in the long-term from a variety of heterogeneous symptoms after the infection. The so-called post-COVID-19 condition (according to the WHO’s case definition) or post-COVID-19 syndrome (according to the NICE guideline on long COVID) occurs in individuals exhibiting symptoms more than three months after the infection. The symptoms can be persistent, fluctuating or relapsing and most commonly include fatigue, dyspnea and sleep disorders. Furthermore, symptoms such as difficulties concentrating, anxiety, depression, effort intolerance, joint pain and myalgia were frequently reported up to more than twelve months after infection [1]. To date, long-term symptoms were even reported up to two years after infection [2,3]. In a recent meta-analysis, considerable (pooled) prevalences were reported for the post-COVID-19 condition, with the prevalence in hospitalized patients being higher (54% (95% CI 44–63%)) than in non-hospitalized patients (34% (95% CI 29–37%)) [4]. There are several risk factors for a post-COVID-19 condition. Women were especially shown to have a significantly greater likelihood of having post-COVID-19 symptoms than men [5]. Other risk factors include (but are not limited to) pre-existing asthma, more severe COVID-19 during the acute phase and older age [4].

Hospitalization seems to be one crucial factor for the presence and the severity of a post-COVID-19 condition. In several systematic reviews, meta-analyses and studies, the condition was compared between hospitalized and non-hospitalized individuals. It was reported that hospitalization led to greater limitations on activities of daily living, had a greater impact on returning to work [6] and increased the risk of dyspnea, anxiety, myalgia and hair loss [7].

However, although symptom prevalence and risk seem to be lower in non-hospitalized individuals in general, the burden of symptoms can be extraordinarily high in individual cases. Severe fatigue, breathlessness and neurocognitive impairment particularly impede the management of day-to-day work and participation in social activities and thus can jeopardize one’s health-related quality of life [8]. According to Tedros Adhanom Ghebreyesus, the director general of the World Health Organization, “long COVID is devastating people’s life and livelihoods” [9]. Patients affected by post-COVID-19 symptoms described disruptions in their work life, social life and home life [10]. According to the results of a qualitative study, individuals with post-COVID-19 conditions mainly experienced a loss of abilities and a loss of control, which led them to re-evaluate their life [11].

Correspondingly, multidisciplinary rehabilitation approaches are recommended for all individuals suffering from post-COVID-19 symptoms [12]. However, waiting lists for COVID-19 specialists and rehabilitation were long [13,14], as the number of people affected by post-COVID-19 symptoms was high, but the number of rehabilitation places and knowledge about effective rehabilitation methods were both scarce.

At the end of 2021 when the burden caused by post-COVID-19 became explicit, a study was initiated by the authors at the Schoen Clinic Bad Aibling. The aim was to develop an interdisciplinary rehabilitation approach for patients with post-COVID-19 conditions [15]. During the recruitment and performance of the study, the extensive severity of symptoms of the study participants became apparent. However, hardly any one of the individuals interested in participating in the study had been hospitalized during the acute COVID-19 disease. The severity of the symptoms was substantial and appeared comparable to the severity experienced by critically ill individuals due to COVID-19 who required intensive care therapy and mechanical ventilation (as we reported previously [16]). However, up to now, scientific investigations about severe post-COVID-19 conditions in non-hospitalized individuals are rare. In many studies it is pronounced that the post-COVID-19 condition is particularly severe and long-lasting in hospitalized individuals [6,7,17]. Additionally, the focus of previous studies was mainly on prevalences of the post-COVID-19 condition, the different manifestations of symptoms and their change over time [4,17]. However, the impact of the post-COVID-19 condition on specific activities of daily living was hardly described. Accordingly, societal knowledge about the possible vast extent of the post-COVID-19 condition is poor, leading to a lack of recognition and understanding of those who are severely affected.

Therefore, the aim of this study was to describe and emphasize the extraordinary severity of post-COVID-19 symptoms in persons with asymptomatic to mild acute COVID-19 infections who were not hospitalized. Furthermore, we aimed to evaluate their physical and mental health and health-related quality of life and to compare them with individuals who suffered from critical illness due to COVID-19. As the post-COVID-19 condition is mainly influenced by gender, we aimed to analyze the symptoms according to gender.

## 2. Materials and Methods

### 2.1. Study Design, Population and Setting

This cross-sectional study is a secondary analysis of two separate studies. All patients were recruited at the Schoen Clinic Bad Aibling, a center for neurological intensive care and inpatient neurorehabilitation in Germany.

The *post-COVID-19 therapy trial* was a quasi-experimental study in which a new rehabilitation intervention for the post-COVID-19 condition was developed and evaluated [15]. The intervention comprised a two-week outpatient therapy (including Nordic Walking, relaxation techniques, breathing training and balance training) at the Schoen Clinic Bad Aibling and an eight-week digital therapy. Adults (≥18 years) with laboratory-confirmed COVID-19 from at least 3 months ago (evaluated by real-time reverse transcriptase PCR) and post-COVID-19 symptoms limiting their general health were eligible for this study. Exclusion criteria were (1) a potentially life-threatening disease preventing participation in an outpatient rehabilitation program, (2) the requirement for inpatient care with supervision by nursing staff and (3) insufficient (German) communication skills to complete the questionnaires and take part in the therapies. This study comprised six study visits in total; for this analysis, only the first study visit (two weeks before the intervention started) was taken into account. By coincidence, nearly all participants had mild acute COVID-19 infections without hospitalization.

The *COVID-19 rehabilitation trial* was an observational prospective cohort study with hospitalized individuals. Adult patients (≥18 years) with laboratory-confirmed COVID-19 (evaluated by real-time reverse transcriptase PCR) were eligible after the infectious stage and after being admitted to neurorehabilitation. Exclusion criteria were (1) insufficient (German) communication skills to complete the questionnaires and (2) patients receiving palliative care. For this analysis, only critically ill COVID-19 patients who received intensive care treatment and invasive mechanical ventilation for >96 h were included. Patients were included at admission to neurological rehabilitation. The five study visits took place at study inclusion; at discharge from neurorehabilitation (both as in-person interviews at the clinic); and 3, 6, and 12 months after discharge (telephone interviews and questionnaires sent by post). In all study visits, a comprehensive set of functional tests, questionnaires and questions about living and working circumstances were performed. For this analysis, only the study visit at 3 months after discharge was taken into account, as this visit took place at approximately the same length of time since the first COVID-19 infection as for the participants in the *post-COVID-19 therapy trial*. Only patients with complete data at this study visit were considered. Part of this study population was described previously in a study on the clinical course during neurorehabilitation and long-term outcomes [16,18].

Both studies were approved by the medical ethics committee of the Ludwig-Maximilians-Universität in Munich according to the Declaration of Helsinki (project no. 22-0310 and 20-0478). Written informed consent was obtained from all participants. The studies were registered at the German Clinical Trials Register (DRKS00029415 and DRKS00025606).

### 2.2. Outcome Measures

The study visits of *the post-COVID-19 therapy trial* were conducted in person; the visits of the *COVID-19 rehabilitation trial* were conducted via telephone interviews and questionnaires sent by post. All study visits were conducted by trained and experienced study staff. 

The following patient-centered outcomes and assessments were used:

- The Fatigue Severity Scale-7 (FSS-7) was used to evaluate fatigue. The seven-item version was utilized, as it has better psychometric properties than the nine-item version [19]. Score: 1–7. The cut-off of ≥4 was interpreted as indicative of fatigue [20].
- The Hospital Anxiety and Depression Scale (HADS) is a valid and reliable tool to measure anxiety and depression and was also previously used in COVID-19 patients [21]. Score: 0–21, each for anxiety and depression. A score of >7 in each category was interpreted as clinically significant [22].
- The EuroQol-5 dimensions-5 level (EQ-5D-5L) was used to measure the health-related quality of life [23]. The index value for the German population ranges from −0.205 (0 = health state equivalent to death; negative values = health state worse than death) to 1.000 (best health state) [24]. Additionally, the visual analogue scale (VAS; included in the EQ-5D-5L; 0–100) was used. A value of 100 indicates the best imaginable state of health.
- The generic World Health Organization Disability Assessment Schedule 2.0 (WHODAS-12) is a measure of disability and functional impairment and comprises the categories cognition, mobility, self-care, getting along, life activities and participation. It is reliable, widely used and has good internal consistency [25]. Each of the twelve scores was scored from 0 (no difficulties) to 4 (extreme difficulties or cannot do). The total score was converted into a percentage ((sum/48) × 100) and allocated to the following groups: no (0–4%); mild (5–24%); moderate (25–49%); severe (50–95%); and complete (96–100%) disability [26].
- To assess dyspnea, the modified medical research council dyspnea scale (mMRC) was used (score 0–4; 4 = severest dyspnea). This scale was repeatedly used in patients with COPD and COVID-19 disease [27,28].

### 2.3. Statistical Analysis

Study participants were selected based on convenience sampling by choosing patients being treated at our clinic (*COVID-19 rehabilitation trial*) or patients who expressed an interest in participating in the *post-COVID-19 therapy trial*. Sample sizes were derived from the number of ICU COVID-19 patients who were treated in our hospital (*COVID-19 rehabilitation trial*) and from pragmatic reasons in terms of limited resources of time, staff and funding (*post-COVID-19 therapy trial)*. 

Categorical variables are presented as absolute values and percentages and continuous variables as mean ± standard deviation or median (quartile 1–quartile 3).

Symptoms were compared between the two study groups and between male and female participants. The Mann–Whitney U test was used when data were either non-parametric or did not follow normal distribution (as checked by the Shapiro–Wilk test and visually by means of Q–Q plots). Independent *t*-tests were applied in cases of parametric data which followed normal distribution. The Chi-squared test was used for categorical values. Fisher’s exact test was applied in cases in which more than 20% of the cells had expected cell counts of less than 5.

Multiple linear regression was performed to show that the health-related quality of life and the degree of disability (WHODAS-12 percentage score) are not predicted by the status of hospitalization (yes/no). To control for confounding, a directed acyclic graph (DAG) was created for the two investigations using DAGitty (https://dagitty.net/, accessed on 04 August 2023). According to the DAG, confounder controlling was conducted for gender, age, vaccination (no, first vaccination and full vaccination) and preclinical comorbidities (Elixhauser comorbidity index). The DAGs with the associated study references can be found in the Supplementary File S1. Effect modification by gender was evaluated by adding a hospitalization-by-gender interaction variable to the regression term and by examining its significance. Assumptions for the multiple linear regression (linearity, multicollinearity, homoscedasticity, multivariate normality and autocorrelation) were tested for systematic violations. 

Statistical analyses were performed using R version 4.1.1 and IBM SPSS Statistics 19. A *p*-value of ≤0.05 was considered significant. Missing data were not replaced.

## 3. Results

**Figure 1:**
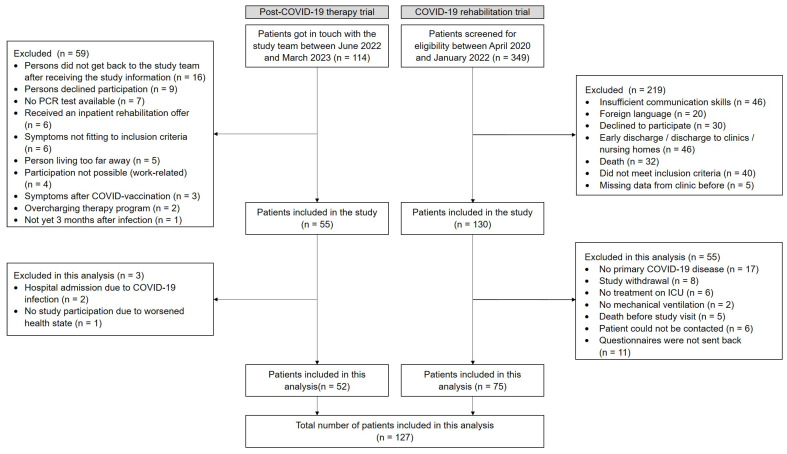
Flow chart for the non-hospitalized individuals (post-COVID-19 therapy trial) and the hospitalized individuals (COVID-19 rehabilitation trial).

In the post-COVID-19 therapy trial (non-hospitalized individuals), 114 individuals were screened between June 2022 and March 2023. A total of 55 were included in the trial, and 52 were enrolled in this analysis. In the COVID-19 rehabilitation trial (hospitalized individuals), 349 patients were screened between April 2020 and January 2022. A total of 130 were enrolled in the study from June 2020 until January 2022, and 75 were included in this analysis (Figure 1). 

The hospitalized patients all had a prolonged stay on the intensive care unit (ICU) (Table 1) and had frequently suffered from sepsis (89.3%), acute respiratory distress syndrome (85.3%) and critical illness polyneuropathy and myopathy (81.4%, as measured by nerve conduction studies). Non-hospitalized individuals were significantly younger, included more females and had significantly fewer prior comorbidities compared to the hospitalized individuals. The duration since the first COVID-19 infection was comparable between the groups and was approximately 7.5 months.

Most of the hospitalized patients were not vaccinated at the time of the SARS-CoV-2 infection, because a high number of patients (n = 36, 48%) were infected during the first two waves in 2020, when a vaccination was not yet available. Another 30 patients were infected during the third wave (until June 2021), when a vaccination was also not yet completely disseminated.

Substantially more individuals in the hospitalized group retired before the infection. The majority of previously employed individuals was on sick leave at the time of the study visits (non-hospitalized: 45%; hospitalized: 59%) or required changes in working hours or working arrangements (non-hospitalized: 27%; hospitalized: 16%). Thus, only a minor group of patients (16–22%) was able to work as before the COVID-19 infection (Table 1). 

Fatigue was more frequently reported by non-hospitalized individuals, and nearly everyone in this group had a fatigue score of >4. The prevalence of anxiety was significantly higher, and there was a tendency for a more pronounced depression among non-hospitalized individuals (Table 2), as displayed by the HADS and the EQ-5D-5L. Accordingly, the emotional affection (as measured by item 5 of the WHODAS-12) was also significantly higher in the non-hospitalized group. The health-related quality of life was reduced in both groups; however, it was more affected in the non-hospitalized individuals.

**Figure 2:**
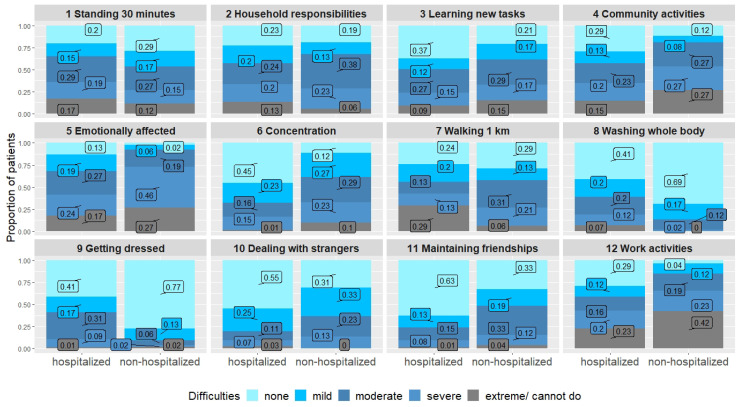
WHODAS-12 comparing hospitalized and non-hospitalized individuals.

Difficulties in basic physical tasks such as washing and getting dressed were more frequently reported by hospitalized persons. However, more exhausting physical tasks such as walking for one kilometer, household responsibilities or standing for longer periods led to similar difficulties in both groups. Dyspnea was comparable in both groups. Concentration was more impaired in the non-hospitalized individuals. Social activities and participation were also more affected in the non-hospitalized group, and work activities and joining in community activities especially led to severe difficulties in this group (Table 3, Figure 2). However, although the burden of symptoms seemed higher in the non-hospitalized group, most of the hospitalized individuals were also substantially impaired, as two thirds had either a moderate or a severe disability (as measured by the WHODAS-12).

Substantial differences in symptoms were found between males and females. The health-related quality of life and overall disability and functional impairment (as measured by WHODAS-12) were significantly lower in both hospitalized and non-hospitalized women. In contrast, no differences were found for fatigue, anxiety, depression and dyspnea (Table 4).

According to the results of the multiple regression analysis, no significant effect of hospitalization on the health-related quality of life and general disability could be shown after accounting for gender, age, vaccination and comorbidities. It was shown that the male gender is significantly associated with a higher health-related quality of life and less general disability (Table 5). No effect modification by gender was found. 

## 4. Discussion

The aim of this analysis was to describe the symptoms of persons with severe post-COVID-19 conditions. Additionally, we evaluated physical and mental health as well as the health-related quality of life and compared them between non-hospitalized individuals with severe post-COVID-19 conditions and individuals after critical illness due to COVID-19. Overall, we demonstrated a high burden of mental and physical symptoms in individuals with severe post-COVID-19 conditions. Persons of both groups mostly suffered from a moderate to severe disability and had a substantially reduced health-related quality of life. However, symptoms such as fatigue, anxiety and difficulties in joining in community activities and work activities were significantly more pronounced in the non-hospitalized individuals. Only gender was found to be significantly associated with health-related quality of life and degree of disability.

### 4.1. Post-COVID-19 Conditions in Non-Hospitalized vs. Hospitalized Individuals

This investigation differs from previous reports. In former studies comparing non-hospitalized and hospitalized COVID-19 patients, patients were distinguished according to the disease severity of the acute infection. In such studies, it was shown that previously hospitalized patients have a higher risk of the post-COVID-19 condition and suffer more frequently from more severe symptoms than non-hospitalized persons [4,6,7,17,29,30].

However, in some investigations no difference in symptoms according to the hospitalization status [3] or even a worse health state in non-hospitalized individuals were reported. The common factor in most of these investigations was that patients who sought support were recruited, e.g., in outpatient or rehabilitation clinics or in COVID-19 support groups. Houben-Wilke et al. (2022) [31] conducted an online survey among Facebook group members (groups for COVID patients with persistent complaints). Six months after the infection, depression and anxiety were reported in 42% and 29% of hospitalized individuals and in 40% and 37% of non-hospitalized individuals. Johnsen et al. (2021) [32] investigated COVID-19 patients three months after discharge from hospital and patients who were referred to a respiratory outpatient clinic by their general practitioner because of persistent post-COVID-19 symptoms. The health related-quality of life was comparable in both groups, although there was a tendency for lower values in the non-hospitalized individuals (median index value of 0.74 (quartile 1 = 0.66-quartile 3 = 0.80) vs. 0.79 (0.65–0.86); VAS 65 (55–79) vs. 75 (59–90)). Perrot et al. (2022) [33] compared post-COVID-19 symptoms in three groups of patients who were admitted to their post-COVID rehabilitation unit (mean duration of 110 days since discharge from the hospital): patients who were not hospitalized, patients admitted to a general ward and patients admitted to the ICU. Anxiety was found to be significantly less frequent in ICU patients (18.7% vs. 40.7–46.7%), and depression was significantly more common in patients who were not admitted to a hospital (37.0% vs. 17.6–26.7%). Accordingly, the mental component of the health-related quality of life questionnaire SF-36 was more reduced in patients who were not treated in the ICU. However, the physical component of the questionnaire did not differ between the three groups, and dyspnea was similarly frequent. Accordingly, the health-related quality of life was significantly deteriorated in all patients.

The higher burden of self-reported symptoms in the non-hospitalized individuals might be due to their different points of view. The non-hospitalized individuals perceived themselves as healthy before and during the infection and now suffer from symptoms. In contrast, the hospitalized patients potentially experienced symptom relief at the time of the study visit, e.g., they were able to breathe independently and live back home again with their families. Additionally, they received close supervision, intensive monitoring and rehabilitation during their hospitalization period. Therefore, the point of view of hospitalized patients might be more positive, while the point of view of non-hospitalized patients might be more negative. This assumption is in line with previous reports [33].

### 4.2. Individuals with Eminently Severe Post-COVID-19 Conditions

Severely affected non-hospitalized participants with symptoms equally severe to those in our particular group of patients have rarely been described so far. Usually, reported symptom prevalences are substantially lower, like in a German population-based study (fatigue, 37%; neurocognitive impairment, 31%; anxiety/depression, 21%) [34]. However, a few studies also reported higher symptom prevalences, similar to our results. For example, Sivan et al. (2021) [35] reported the severity of symptoms of 370 mainly non-hospitalized patients recruited in a dedicated community COVID-19 rehabilitation service. The burden of symptoms (at a median duration of 211 days after infection) was extraordinarily high, as 95% of the patients reported fatigue, 90% reported anxiety, 89% reported pain or discomfort, 85% reported breathlessness and 85% reported cognitive deficits. Tabacof et al. (2022) described the health state of 156 patients (89% initially not hospitalized) who were recruited at an interdisciplinary clinic for post-acute COVID-19 syndrome with a median of 351 days after the infection. A median fatigue score of 5.6 and problematic fatigue (score > 4) were observed in 78% of the patients (as indicated by the Fatigue Severity Scale as in our study). Although fatigue was comparable to our results, and other symptoms such as brain fog (67%), headache (60%) and sleep disturbances (59%) were frequently reported, the frequency of anxiety (19%) and depression (28%) were much lower, and the health-related quality of life was higher (median VAS 64) compared to our two groups of patients.

A reason for the extraordinarily high frequency of symptoms in our group of non-hospitalized patients might be that patients actively got in touch with the study team to participate in the therapy trial due to their high burden of symptoms. This special selection of participants allows for a detailed consideration of patients requiring intensive support and therapies, a group that can easily be overlooked in population studies.

### 4.3. Gender Inequality of the Post-COVID-19 Condition

In previous studies, it was repeatedly shown that women have a higher risk of developing post-COVID symptoms [5,36]. Fatigue, dyspnea, mental health issues and sleep disturbances were especially more frequently reported in women [5,36,37,38]. Accordingly, the health-related quality of life was found to be more reduced in women [37]. Our findings are partially in line with these reports. The burden of symptoms seems to be higher in our participating women, as their WHODAS-12 and health-related quality of life scores were significantly more reduced compared to those of the men. Additionally, fatigue and depression were more frequent in women. In contrast, anxiety and dyspnea were equally reported by the male and female participants. This could be either a result of an insufficient sample size or a result of a selection bias in the participants of the post-COVID therapy trial (as outlined in the limitation section). However, the high number of men affected by anxiety (68% of non-hospitalized persons, 42% of hospitalized persons) should be noted, as this indicates a great need for assistance and therapy—not only for women.

### 4.4. Rehabilitation Recommendations

The reported severity of symptoms, the high percentages of sick leave and reduced working capacities and the negative impact on social life highlight the importance of tailored rehabilitation services. As we confirmed that the manifestation and severity of symptoms can occur irrespective of the severity of the acute illness, the “prescription and provision of rehabilitation programs should be guided by persistent symptoms and functional limitations”, as it was stated by the WHO [39]. Individuals suffering mainly from fatigue and mental health issues (like our group of non-hospitalized patients) require approaches like education and skills training on energy conservation techniques and psychological support [39]. In contrast, patients suffering mainly from functional impairments (like our hospitalized patients) and breathing impairments may more likely benefit from education and skills training on self-management strategies for breathing techniques and physical exercise training [39]. However, these recommendations are based on a low certainty of evidence, and high-quality studies are urgently needed to provide beneficial therapy approaches for individuals suffering from long-term persisting symptoms after COVID-19 [40].

### 4.5. Limitations

There are a number of limitations to our approach. For the non-hospitalized group, only those persons were included who actively applied to take part in our outpatient rehabilitation study. This certainly causes a selection bias. For example, only those individuals with severe symptoms but enough mental confidence, those that were able to come to the clinic, had a sick note or had enough freedom regarding family commitments would apply to participate. Additionally, fatigue might be one of the severest symptoms with the highest impact on normal living. Therefore, it is not surprising that fatigue occurred so frequently in our group of non-hospitalized individuals who sought support. However, we did not intend to give a representative sample but rather aimed to compare two highly affected groups.

Furthermore, our group of hospitalized COVID-19 patients is also very special. In this group, very severely affected patients with extraordinary long durations of ICU treatment and mechanical ventilation are represented [41,42]. As health limitations are common in COVID-19 ICU survivors even after short durations of ICU therapy [43,44], severe and enduring health deficits seem likely in critically ill COVID-19 patients requiring substantially longer times of intensive care treatment [45,46].

Additionally, a larger sample size would have been of advantage to emphasize the concomitant societal burdens and challenges. Therefore, our results are not generalizable regarding hospitalized or non-hospitalized COVID-19 patients in general.

Another limitation is the cross-sectional design of this study, which does not allow for the presence of symptoms to be attributed solely to the infection of SARS-CoV-2. In addition, we did not evaluate the different SARS-CoV-2 variants which might be of relevance, as there were differences in the occurrence, length and severity of the different variants [47].

## 5. Conclusions

We reported two cohorts with a high burden of post-COVID-19 conditions including mental and physical symptoms as well as a limited health-related quality of life. We observed a moderate to severe disability in both groups. However, symptoms such as fatigue, anxiety and difficulties in joining in community activities and work activities were significantly more pronounced in the non-hospitalized individuals. The female gender, but not hospitalization, was found to be significantly associated with the health-related quality of life and degree of disability. This study emphasizes the severity of post-COVID-19 conditions (even after mild acute infections), its high impact on the daily living of those affected and the need for individualized follow-up services and treatments.

## Figures and Tables

**Table 1:** Characteristics of the participants.

	Non-Hospitalized Individuals (n = 52)	Hospitalized Individuals (n = 75)
Age at first COVID-19 infection, years	46.9 ± 15.6	61.4 ± 12.1 *
Sex, women	33 (63.5%)	22 (29.3%) ^†^
Duration since first SARS-CoV-2 infection, days	235 (161–412)	214 (178–242) ^‡^
Length of ICU stay, days	NA	49 (36–66)
Length of mechanical ventilation, days	NA	37 (22–52)
Length of hospitalization, days	NA	107 (78–133)
Extracorporeal membrane oxygenation	NA	16 (21.3%)
Elixhauser Comorbidity Index	0.3 ± 1.8	3.6 ± 6.6 *
Comorbidities		
Diabetes (all type II)	2 (3.8%)	16 (21.3%) ^†^
Obesity	3 (5.8%)	18 (24.0%) ^†^
Hypertension	6 (11.5%)	35 (46.7%) ^†^
Vaccination status at first SARS-CoV-2 infection		
No COVID-19 vaccination	18 (34.6%)	67 (90.5%) ^§^
First COVID-19 vaccination	3 (5.8%)	6 (8.0%)
Full COVID-19 vaccination	28 (53.8%)	1 (1.3%)
Missing	3 (5.8%)	1 (1.3%)
Occupation affected by COVID-19		
Retired before COVID-19	3 (5.8%)	29 (38.7%)
Missing data	-	2 (2.7%)
Subgroup of persons being employed before	49 (100%)	44 (100%) ^¶^
Employed—no change	11 (22.4%)	7 (15.9%)
Employed—on reduced working hours or changes in working arrangements	13 (26.5%)	7 (15.9%)
Employed—on sickness leave	22 (44.9%)	26 (59.1%)
Employed—had to retire/change job	0 (0.0%)	4 (9.1%)
Employed—lost job	3 (5.8%)	0 (0.0%)

* *p* < 0.001, as tested with independent *t*-tests; ^†^ *p* < 0.008, as tested with Chi-squared test; ^‡^ *p* = 0.165, as tested with Mann—Whitney U test (as data did not follow normal distribution); ^§^ *p* < 0.001, as tested with Fisher’s exact test; ^¶^ *p* = 0.042, as tested with Fisher’s exact test.

**Table 2:** Fatigue, mental health and health related-quality of life of non-hospitalized vs. hospitalized individuals.

	Non-Hospitalized Individuals (n = 52)	Hospitalized Individuals (n = 75)	*p*-Value
FSS-7	5.6 ± 1.3	3.8 ± 2.0	<0.001 ^‡^
Fatigue ≥4	45 (86.5%)	34 (45.3%)	<0.001 *
HADS			
Anxiety	10 (6–12)	7 (3–10)	0.001 ^†^
Anxiety >7	36 (69.2%)	32 (42.7%)	0.004 *
Depression	8 (5–10)	6 (3–10)	0.064 ^†^
Depression >7	29 (55.8%)	32 (42.7%)	0.154 *
EQ-5D-5L			
Visual Analogue Scale	46.7 ± 20.2	56.5 ± 21.3	0.010 ^‡^
Index value	0.62 ± 0.23	0.68 ± 0.25	0.130 ^‡^
Problems with walking around	2 (1–3); 29 (53.8%)	2 (1–4); 56 (74.7%)	0.055 ^†^
Problems with washing/dressing	1 (1–1); 12 (23.1%)	1 (1–3); 36 (48.0%)	0.004 ^†^
Problems with usual activity	3 (2–4); 43 (82.7%)	2 (2–4); 60 (80.0%)	0.114 ^†^
Pain or discomfort	4 (3–4); 49 (94.2%)	3 (2–3); 62 (82.7%)	<0.001 ^†^
Anxiety or depression	2 (2–4); 40 (76.9%)	2 (1–3); 39 (52.0%)	0.002 ^†^
Dyspnea	1 (0–2)	1 (0–2)	0.623 ^†^
No dyspnea (MMRC Score 0)	14 (26.9%)	17 (34.0%) ^¶^	0.437 *

Data are n (%), mean ± SD or median (quartile 1–quartile 3); FSS-7 = Fatigue-Severity-Scale-7; HADS = Hospital Anxiety and Depression Scale; EQ-5D-5l = EuroQol–5 dimensions–5 level; WHODAS-12 = World Health Organization Disability Assessment Schedule 2.0–12 items; ^†^ Mann–Whitney U test; ^‡^ independent *t*-test; * Chi-squared test; ^¶^ only n = 50 scores available.

**Table 3:** WHODAS-12 in non-hospitalized vs. hospitalized individuals.

	Non-Hospitalized Individuals (n = 52)	Hospitalized Individuals (n = 75)	*p*-Value
WHODAS-12 percentage score	0.43 ± 0.17	0.38 ± 0.24	0.153 ^‡^
WHODAS-12 percentage group			0.053 *
No disability	-	8 (10.7%)	
Mild disability	9 (17.3%)	17 (22.7%)	
Moderate disability	25 (48.1%)	25 (33.3%)	
Severe disability	18 (34.6%)	25 (33.3%)	
Complete disability	-	-	
1. Standing for long periods	2 (0–3)	2 (1–3)	0.153 ^†^
2. Household responsibilities	2 (1–3)	2 (1–3)	0.894 ^†^
3. Learning new tasks	2 (1–3)	2 (0–2)	0.090 ^†^
4. Joining in community activities	3 (2–4)	2 (0–3)	0.005 ^†^
5. Emotionally affected	3 (2–4)	2 (1–3)	0.001 ^†^
6. Concentrating for 10 min	2 (1–3)	1 (0–2)	<0.001 ^†^
7. Walking a long distance (1 km)	2 (1–3)	2 (1–4)	0.122 ^†^
8. Washing one’s whole body	0 (0–1)	1 (0–2)	<0.001 ^†^
9. Getting dressed	0 (0–0)	1 (0–2)	<0.001 ^†^
10. Dealing with strangers	1 (0–2)	0 (0–1)	0.009 ^†^
11. Maintaining friendships	1 (0–2)	0 (0–1)	0.001 ^†^
12. Work/school activities	3 (2–4)	2 (0–3)	0.001 ^†^

Data are n (%), mean ± SD or median (quartile 1–quartile 3); WHODAS-12 = World Health Organization Disability Assessment Schedule 2.0–12 items; ^†^ Mann–Whitney U test; ^‡^ independent *t*-test; * Chi-squared test.

**Table 4:** Post-COVID-19 symptoms by gender and hospitalization status.

	Non-Hospitalized Individuals (n = 52)			Hospitalized Individuals (n = 75)		
	Female (n = 33)	Male (n = 19)	*p*-Value	Female (n = 22)	Male (n = 53)	*p*-Value
FSS-7	5.9 ± 1.2	5.1 ± 1.4	0.030 ^‡^	4.2 ± 2.0	3.6 ± 2.0	0.237 ^‡^
Fatigue ≥4	30 (90.9%)	15 (78.9%)	0.400 *	12 (54.5%)	22 (41.5%)	0.302 *
HADS						
Anxiety	10 (6–14)	10 (6–12)	0.561 ^†^	7 (4–12)	6 (3–10)	0.327 ^†^
Anxiety >7	23 (69.7%)	13 (68.4%)	0.924 *	10 (45.5%)	22 (41.5%)	0.753 *
Depression	8 (7–10)	7 (5–10)	0.184 ^†^	9 (3–11)	6 (3–9)	0.353 ^†^
Depression >7	21 (63.6%)	8 (42.1%)	0.132 *	12 (54.5%)	20 (37.7%)	0.180 *
EQ-5D-5L						
VAS	41.6 ± 19.0	55.5 ± 19.5	0.015 ^‡^	47.7 ± 25.0	60.2 ± 18.5	0.020 ^‡^
Index value	0.55 ± 0.23	0.73 ± 0.17	0.003 ^‡^	0.60 ± 0.31	0.72 ± 0.22	0.105 ^‡^
Dyspnea	1 (0–2)	1 (0–2)	0.646 ^†^	1 (0–2)	1 (0–2)	0.831 ^†^
WHODAS-12 percentage score	0.48 ± 0.14	0.34 ± 0.18	0.002 ^‡^	0.47 ± 0.27	0.34 ± 0.23	0.037 ^‡^

Data are n (%), mean ± SD or median (quartile 1–quartile 3); FSS-7 = Fatigue-Severity-Scale-7; HADS = Hospital Anxiety and Depression Scale; EQ-5D-5l = EuroQol–5 dimensions–5 level; VAS = Visual Analogue Scale (0–100); WHODAS-12 = World Health Organization Disability Assessment Schedule 2.0–12 items; ^†^ Mann–Whitney U test; ^‡^ independent *t*-test; * Chi-squared test or Fisher’s exact test.

**Table 5:** Results of the multiple linear regression.

	Health Related Quality of Life—Visual Analogue Scale		WHODAS-12 Percentage Score—General Disability	
	Estimate	95% CI	Estimate	95% CI
Intercept	41.33 ***	23.39–59.27	0.42 ***	0.23–0.60
Hospitalization = none	−5.06	−16.54–6.42	−0.00	−0.12–0.12
Gender = male	11.79 **	3.70–19.88	−0.16 ***	−0.24–−0.07
Age	0.12	−0.16–0.40	0.00	−0.00–0.00
First Vaccination	5.18	−10.01–20.37	0.09	−0.06–0.24
Full Vaccination	0.98	−10.53–12.49	0.05	−0.07–0.17
Comorbidities	−0.15	−0.88–0.58	0.00	−0.01–0.01

*** < 0.001; ** < 0.01; df = degrees of freedom; Statistical values: Health-related quality of life—R^2^ = 0.14, adjusted R^2^ = 0.10; F-statistic (degrees of freedom)—(6,115) = 3.23 **; WHODAS-12—R^2^ = 0.13, adjusted R^2^ = 0.08, F(6,116) = 2.79 *.

## Data Availability

The datasets used and/or analyzed during the current study are available from the corresponding author on reasonable request.

## Supplementary Materials

The following supporting information can be downloaded at: https://www.mdpi.com/article/10.3390/ijerph21010021/s1, Supplement S1: DAGs and supporting literature.
