# Supplementary material for: Severe Post-COVID-19 Condition after Mild Infection: Physical and Mental Health Eight Months Post Infection: A Cross-Sectional Study
Source: Int J Environ Res Public Health. 2023 Dec 22;21(1):21. doi: 10.3390/ijerph21010021 (PMC10815598; doi:10.3390/ijerph21010021)
Supplement: Supplementary file 1 [file ijerph-21-00021-s001.zip › ijerph-2684264-supplementary.pdf]

Supplement S1 – DAGs and supporting literature

Severe Post COVID-19 Condition after Mild Infection: Physical and Mental Health Eight Months Post Infection: A Cross-Sectional Study

Authors: Marion Egger, Lena Vogelgesang, Judith Reitelbach, Jeannine Bergmann, Friedemann Müller, Klaus Jahn

Corresponding author: Marion Egger, megger@schoen-klinik.de

Literature for the DAGs:

- [1] Negative influence of fatigue on health-related quality of life (in ICU survivors) Self-reported fatigue following intensive care of chronically critically ill patients: a prospective cohort study | Journal of Intensive Care | Full Text (biomedcentral.com)
- [2] Fatigue is more frequent in persons with more preexisting medical comorbidities (Post–COVID-19 Symptoms 2 Years After SARS-CoV-2 Infection Among Hospitalized vs Nonhospitalized Patients | Infectious Diseases | JAMA Network Open | JAMA Network)
- [3] Fatigue is more frequent in women (Biomedicines | Free Full-Text | Trajectory of Post-COVID Self-Reported Fatigue and Dyspnoea in Individuals Who Had Been Hospitalized by COVID-19: The LONG-COVID-EXP Multicenter Study (mdpi.com)); Fatigue after COVID-19 in non-hospitalized patients according to sex - PubMed (nih.gov)
- [4] Fatigue is more frequent in hospitalized patients (Long COVID in hospitalized and non-hospitalized patients in a large cohort in Northwest Spain, a prospective cohort study | Scientific Reports (nature.com))
- [5] Depression has a negative influence on HRQL in ICU survivors Quality of Life, Depression, and Anxiety in Survivors of Critical Illness from a Greek ICU. A Prospective Observational Study - PMC (nih.gov)
- [6] Anxiety has a negative influence on HRQL in ICU survivors (Frontiers | The effect of anxiety and depression on the health-related quality of life of severe acute pancreatitis survivors: structural equation modeling approach (frontiersin.org))
- [7] Women have a lower HRQL than men (Gender differences in health-related quality of life in patients undergoing coronary angiography | Open Heart (bmj.com); Health-related quality of life of COVID-19 patients after discharge: A multicenter follow-up study - PubMed (nih.gov))
- [8] Anxiety is more frequent in women (Gender Effects on Depression, Anxiety, and Stress Regarding the Fear of COVID-19 - PMC (nih.gov))
- [9] Comorbidities influence HRQL (Health-related quality of life in intensive care survivors: Associations with social support, comorbidity, and pain interference | PLOS ONE)
- [10] Comorbidities are a risk factor for anxiety (Prevalence and risk factors of COVID-19-related generalized anxiety disorder among the general public in China: a cross-sectional study - PMC (nih.gov))

- [11] Age negatively influences anxiety and depression (Psych | Free Full-Text | Risk Factors and Changes in Depression and Anxiety over Time in New Zealand during COVID-19: A Longitudinal Cohort Study (mdpi.com))
- [12] Fatigue risk factors are old age, female gender, preclinical comorbidities, severe acute infection (Frontiers | Post-COVID-19 fatigue: A systematic review (frontiersin.org))
- [13] Risk factors for a decline in HRQL are gender, age, severe acute infection, preclinical comorbidity Trajectories of health-related quality of life and their predictors in adult COVID-19 survivors: A longitudinal analysis of the Biobanque Québécoise de la COVID-19 (BQC-19) - PMC (nih.gov)
- [14] Gender is a risk factor for depression (COVID-19 depression and its risk factors in Asia Pacific – A systematic review and meta-analysis - ScienceDirect)
- [15] Risk factors for functional impairment: gender, oxygen therapy during acute disease Risk factors and severity of functional impairment in long COVID: a single-center experience in Croatia - PMC (nih.gov)
- [16] Severity of acute disease influences the degree of function impairments / post-COVID-19 condition Impairments following COVID-19 infection: manifestations and investigations of related factors | Scientific Reports (nature.com); Risk factors for impaired respiratory function post COVID-19: A prospective cohort study of nonhospitalized and hospitalized patients - PubMed (nih.gov)
- [17] Fatigue increases depression Prevalence, trajectory over time, and risk factor of post-COVID-19 fatigue - PMC (nih.gov); Mental health outcomes following COVID-19 infection: impacts of post-COVID impairments and fatigue on depression, anxiety, and insomnia — a web survey in Sweden | BMC Psychiatry | Full Text (biomedcentral.com)
- [18] Fatigue increases anxiety Mental health outcomes following COVID-19 infection: impacts of post-COVID impairments and fatigue on depression, anxiety, and insomnia — a web survey in Sweden | BMC Psychiatry | Full Text (biomedcentral.com)
- [19] Body impairments increase anxiety and depression Mental health outcomes following COVID-19 infection: impacts of post-COVID impairments and fatigue on depression, anxiety, and insomnia — a web survey in Sweden | BMC Psychiatry | Full Text (biomedcentral.com)
- [20] Less post-COVID 19 symptoms after vaccination (Trajectory of long covid symptoms after covid-19 vaccination: community based cohort study - PubMed (nih.gov)), Impact of COVID-19 vaccination on the risk of developing long-COVID and on existing long-COVID symptoms: A systematic review - PubMed (nih.gov), Effect of covid-19 vaccination on long covid: systematic review - PubMed (nih.gov)
- [21] Vaccination leads to less anxiety and depression Mental health outcomes after SARS-CoV-2 vaccination in the United States: A national cross-sectional study - PubMed (nih.gov)
- [22] Vaccines reduce severe or critical disease courses Efficacy and safety of COVID-19 vaccines - PubMed (nih.gov)
- [23] Anxiety and Depression decrease over time Trajectory curves of post-COVID anxiety/depressive symptoms and sleep quality in previously hospitalized COVID-19 survivors: the LONG-COVID-EXP-CM multicenter study | Psychological Medicine | Cambridge Core
- [24] Fatigue decreases over time Biomedicines | Free Full-Text | Trajectory of Post-COVID Self-Reported Fatigue and Dyspnoea in Individuals Who Had Been Hospitalized by COVID-19: The LONG-COVID-EXP Multicenter Study (mdpi.com)
- [25] Post-COVID symptoms improve over time Trajectories of the evolution of post-COVID-19 condition, up to two years after symptoms onset - ScienceDirect

- [26] Old age, comorbidities and male gender are risk factors for more severe COVID-19 diseases Frontiers | Gender Differences in Patients With COVID-19: Focus on Severity and Mortality (frontiersin.org)
- [27] Multimorbidity is associated with functional decline Functional decline and associated factors in patients with multimorbidity at 8 months of follow-up in primary care: the functionality in pluripathological patients (FUNCIPLUR) longitudinal descriptive study - PMC (nih.gov)
- [28] Differing patterns of multimorbidity by gender, with greater functional impairment in women and more comorbidity in men Multimorbidity gender patterns in hospitalized elderly patients - PMC (nih.gov)
- [29] Depression is more likely in persons with multimorbidity Multimorbidity and depression: A systematic review and meta-analysis - ScienceDirect
- [30] More anxiety in hospitalized COVID-19 patients Frontiers | Post-acute COVID-19 symptom risk in hospitalized and non-hospitalized COVID-19 survivors: A systematic review and meta-analysis (frontiersin.org)
- [31] More and longer hosiptalization lead to more depression Hospitalization and other risk factors for depressive and anxious symptoms in oncological and non-oncological patients - PubMed (nih.gov)
- [32] Functional impairment and decline are common in middle age Functional Impairment and Decline in Middle Age: A Cohort Study - PubMed (nih.gov)
- [33] Fatigue is negatively correlated to health-related quality of life (and the physical component of theSF-36) Quality of Life, Fatigue, and Physical Symptoms Post-COVID-19 Condition: A Cross-Sectional Comparative Study - PMC (nih.gov)
- [34] Functional impairments / disability is related to decreased health-related quality of life Correlation between functional disability and quality of life among rural elderly in Anhui province, China: a cross-sectional study | BMC Public Health | Full Text (biomedcentral.com)
- [35] COVID-19 vaccination advise for persons with comorbidities COVID-19 Vaccines Advice (who.int)
- [36] COVID-19 vaccination reduces the risk for long-COVID Impact of COVID-19 vaccination on the risk of developing long-COVID and on existing long-COVID symptoms: A systematic review - eClinicalMedicine (thelancet.com)

DAG for health-related quality of life:

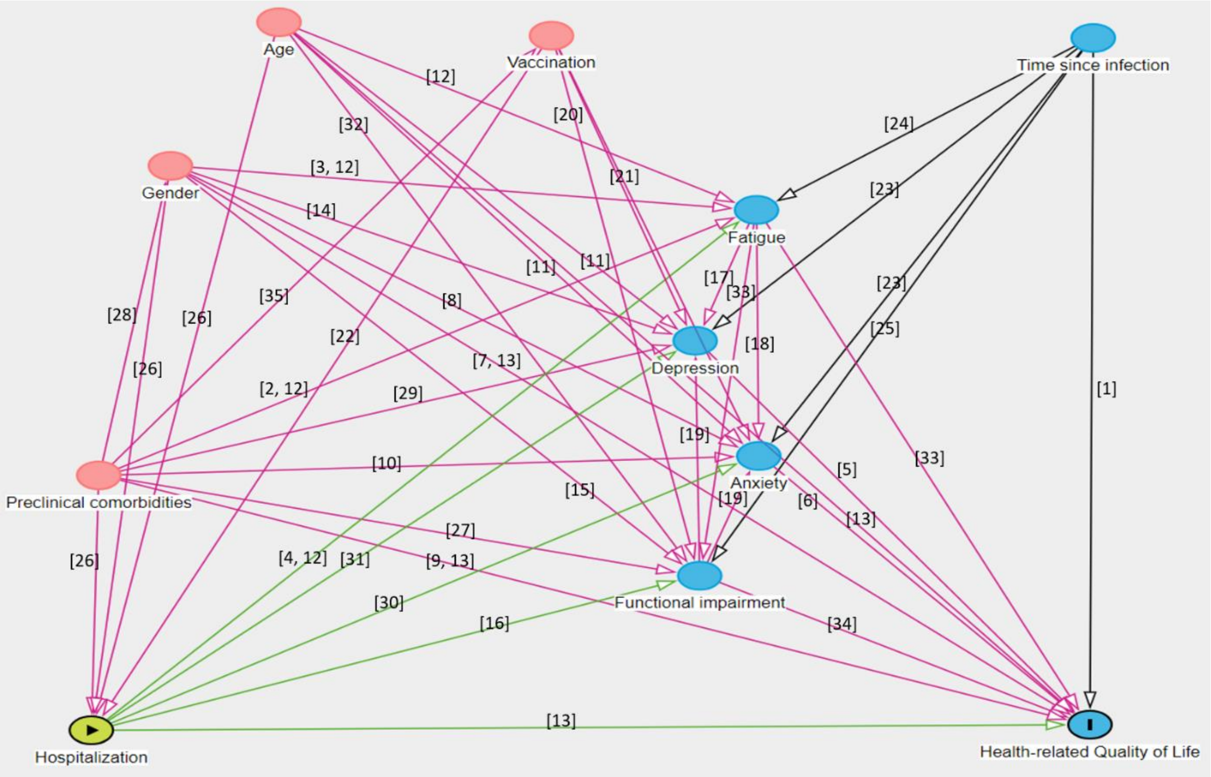

dag {

"Functional impairment" [pos="-0.647,0.484"]

"Health-related Quality of Life" [outcome,pos="0.040,1.011"]

"Preclinical comorbidities" [pos="-1.705,0.129"]

"Time since infection" [pos="0.046,-1.420"]

Age [pos="-1.305,-1.479"]

Anxiety [pos="-0.543,0.060"]

Depression [pos="-0.655,-0.348"]

Fatigue [pos="-0.547,-0.811"]

Gender [pos="-1.579,-0.967"]

Hospitalization [exposure,pos="-1.718,1.031"]

Vaccination [pos="-0.908,-1.428"]

"Functional impairment" -> "Health-related Quality of Life"

"Functional impairment" -> Anxiety

"Functional impairment" -> Depression

"Preclinical comorbidities" -> "Functional impairment"

"Preclinical comorbidities" -> "Health-related Quality of Life"

"Preclinical comorbidities" -> Anxiety

"Preclinical comorbidities" -> Depression

"Preclinical comorbidities" -> Fatigue

"Preclinical comorbidities" -> Gender

"Preclinical comorbidities" -> Hospitalization

"Preclinical comorbidities" -> Vaccination

"Time since infection" -> "Functional impairment"

"Time since infection" -> "Health-related Quality of Life"

"Time since infection" -> Anxiety

"Time since infection" -> Depression

"Time since infection" -> Fatigue

Age -> "Functional impairment"

Age -> Anxiety

Age -> Depression

Age -> Fatigue

Age -> Hospitalization

Anxiety -> "Health-related Quality of Life"

Depression -> "Health-related Quality of Life"

Fatigue -> "Functional impairment"

Fatigue -> "Health-related Quality of Life"

Fatigue -> Anxiety

Fatigue -> Depression

Gender -> "Functional impairment"

Gender -> "Health-related Quality of Life"

Gender -> Anxiety

Gender -> Depression

Gender -> Fatigue

Gender -> Hospitalization

Hospitalization -> "Functional impairment"

Hospitalization -> "Health-related Quality of Life"

Hospitalization -> Anxiety

Hospitalization -> Depression

Hospitalization -> Fatigue

Vaccination -> "Functional impairment"

Vaccination -> Anxiety

Vaccination -> Depression

Vaccination -> Hospitalization

}

DAG für WHODAS / Functional impairment:

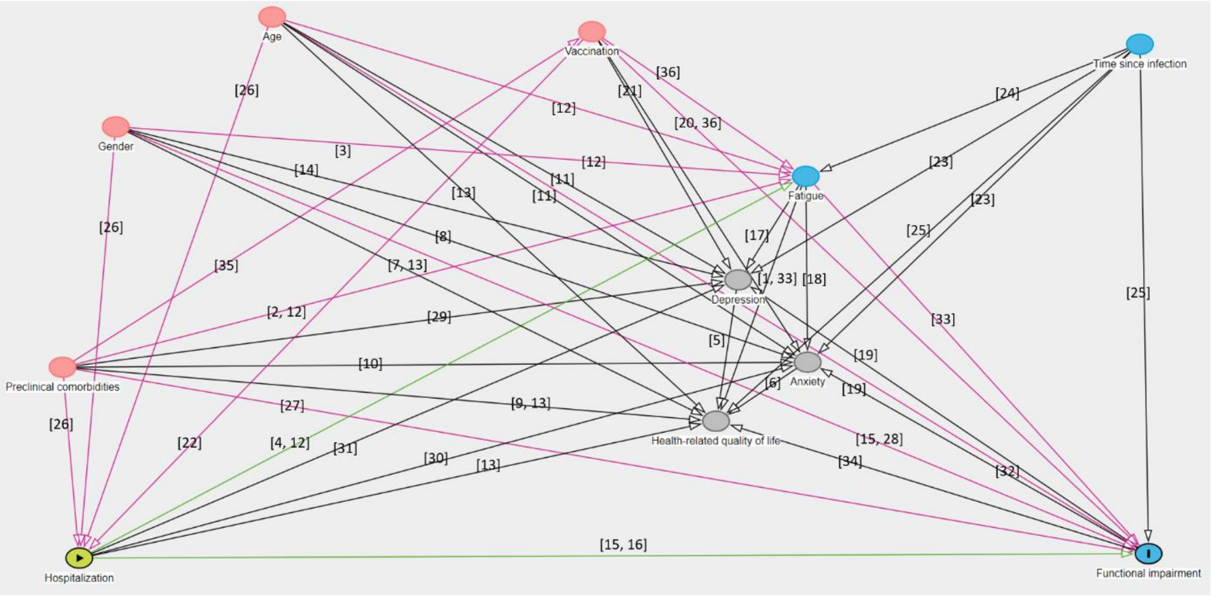

dag {

"Functional impairment" [outcome,pos="0.007,1.009"]

"Health-related quality of life" [pos="-0.691,0.352"]

"Preclinical comorbidities" [pos="-1.745,0.085"]

"Time since infection" [pos="-0.007,-1.516"]

Age [pos="-1.407,-1.651"]

Anxiety [pos="-0.543,0.060"]

Depression [pos="-0.655,-0.348"]

Fatigue [pos="-0.546,-0.861"]

Gender [pos="-1.659,-1.106"]

Hospitalization [exposure,pos="-1.718,1.031"]

Vaccination [pos="-0.891,-1.578"]

"Functional impairment" -> "Health-related quality of life"

"Functional impairment" -> Anxiety

"Functional impairment" -> Depression

"Preclinical comorbidities" -> "Functional impairment"

"Preclinical comorbidities" -> "Health-related quality of life"

"Preclinical comorbidities" -> Anxiety

"Preclinical comorbidities" -> Depression

"Preclinical comorbidities" -> Fatigue

"Preclinical comorbidities" -> Hospitalization

"Preclinical comorbidities" -> Vaccination

"Time since infection" -> "Functional impairment"

"Time since infection" -> "Health-related quality of life"

"Time since infection" -> Anxiety

"Time since infection" -> Depression

"Time since infection" -> Fatigue

Age -> "Functional impairment"

Age -> "Health-related quality of life"

Age -> Anxiety

Age -> Depression

Age -> Fatigue

Age -> Hospitalization

Anxiety -> "Health-related quality of life"

Depression -> "Health-related quality of life"

Fatigue -> "Functional impairment"

Fatigue -> "Health-related quality of life"

Fatigue -> Anxiety

Fatigue -> Depression

Gender -> "Functional impairment"

Gender -> "Health-related quality of life"

Gender -> Anxiety

Gender -> Depression

Gender -> Fatigue

Gender -> Hospitalization

Hospitalization -> "Functional impairment"

```
Hospitalization -> "Health-related quality of life"
Hospitalization -> Anxiety
Hospitalization -> Depression
Hospitalization -> Fatigue
Vaccination -> "Functional impairment"
Vaccination -> Anxiety
Vaccination -> Depression
Vaccination -> Fatigue
Vaccination -> Hospitalization
}
```
